# A 4 bp InDel in the Promoter of Wheat Gene *TaAFP-B* Affecting Seed Dormancy Confirmed in Transgenic Rice

**DOI:** 10.3389/fpls.2022.837805

**Published:** 2022-03-31

**Authors:** Yumei Feng, Yang Han, Bing Han, Yongying Zhao, Yan Yang, Yanping Xing

**Affiliations:** ^1^Key Laboratory of Germplasm Innovation and Utilization of Triticeae Crops at Universities of Inner Mongolia Autonomous Region, College of Life Sciences, Inner Mongolia Agricultural University, Hohhot, China; ^2^Institute of Grassland Research, Chinese Academy of Agricultural Sciences, Hohhot, China; ^3^Henan Key Laboratory of Wheat Biology, National Engineering Laboratory for Wheat, Key Laboratory of Wheat Biology and Genetic Breeding in Central Huang-Huai Region, Ministry of Agriculture and Rural Affairs, Wheat Research Institute, Henan Academy of Agricultural Sciences, Zhengzhou, China

**Keywords:** agrobacterium-mediated transformation, allelic variation, InDel, pre-harvest sprouting, wheat

## Abstract

**Background:**

Wheat (*Triticum aestivum* L.) ABA insensitive five (*ABI5*) binding protein gene (*TaAFP*) is a homologue of the *ABI5* binding protein (AFP) gene in *Arabidopsis thaliana*. It is well documented that *AtAFP* is a negative regulator of ABA signaling that regulates embryo germination and seed dormancy. *TaABI5* was earlier shown to be expressed specifically in seed and its transcript accumulated during wheat grain maturation and acquisition of dormancy. It plays an important role in seed dormancy. In a previous study, we identified two allelic variants *TaAFP-B1a* and *TaAFP-B1b* of *TaAFP* on chromosome arm 2BS in common wheat, designated as, respectively. Sequence analysis revealed a 4 bp insertion in the promoter of *TaAFP-B1a* compared with *TaAFP-B1b* that affected mRNA transcription level, mRNA stability, GUS and tdTomatoER translation level, and GUS activity determining seed dormancy.

**Results:**

The transcription and translation levels of *TaAFP-B* were significantly reduced in *TaAFP-Ba* and *TaAFP-Ba-GFP* transgenic plants compared with *TaAFP-Bb* and *TaAFP-Bb-GFP*. The average GI (germination index) values of *TaAFP-Ba* and *TaAFP-Ba-GFP* were significantly lower than those of *TaAFP-Bb* and *TaAFP-Bb-GFP* in T1 and T2 transgenic rice seeds, whereas mature *TaAFP-Ba* and *TaAFP-Ba-GFP* transgenic seeds exhibited increased ABA sensitivity and content of endogenous ABA compared with *TaAFP-Bb* and *TaAFP-Bb-GFP*.

**Conclusion:**

The 4 bp insertion in the promoter of *TaAFP-Ba* decreased transcript abundance and translation level in transgenic rice. This insertion increased sensitivity to ABA and content of endogenous ABA in mature seeds, leading to a higher seed dormancy and pre-harvest sprouting tolerance in transgenic rice.

## Background

Pre-harvest sprouting (PHS) is a worldwide problem in wheat production. It causes significant losses in grain weight and reduced end-use quality (Groos et al., 2002; Humphreys and Noil, 2002). Generally, PHS decreases grain yield by 6–10%, and the value sprouted wheat can be reduced by 20–50% (Zhang et al., 2017). Severely sprouted wheat cannot be used for flour production or other applications in the food industry and therefore can only be used for animal feed (Simsek et al., 2014). Seed dormancy is the major factor reducing the risk of PHS under wet weather conditions (Li et al., 2004). Therefore, it is important to understand the genetic mechanism of seed dormancy in wheat.

The balance of abscisic acid (ABA) and gibberellin (GA) levels is a major regulator of seed dormancy in plants. ABA regulates germination and promotes mature seed dormancy by inhibiting α-amylase synthesis (Hoffmann, 1997). The mechanism of ABA sensitivity in seeds has been extensively studied in *Arabidopsis*. Several genes associated with seed dormancy have been identified as factors involved in ABA signaling and ABA biosynthesis (Lee and Kende, 2001; Oikawa et al., 2004; Liu et al., 2007; Sugimoto et al., 2010). The ABA signaling pathway includes transcription factors that serve as both positive and negative regulators. The positive regulatory factors are *viviparous-1* (*Vp-1*), *ABI3*, *ABI4*, and *ABI5* (Finkelstein, 1994; Finkelstein and Lynch, 2000). In ABA signaling, *ABI3* and *ABI5* act as intermediates, regulating the maturation and germination of seeds; expression of these genes facilitates desiccation tolerance of seeds at later stages of maturity (Lopez-Molina et al., 2001, 2002; Carles et al., 2002). *ABI3*, *ABI4*, and *ABI5* also affect seed development and ABA sensitivity. A null mutation of *abi3* conferred a more severe effect than that of *abi4* or *abi5* (Parcy et al., 1994; Finkelstein et al., 1998; Finkelstein and Lynch, 2000). Negative regulators include ABI1, ABI2, AIP2 E3 ligase, RING E3 ligase, and ABA insensitive five binding protein (AFP) that regulates ABI5 (Gosti et al., 1999; Lopez-Molina et al., 2003; Stone et al., 2006). *ABI3* is required for appropriate expression of *ABI5* (Finkelstein and Lynch, 2000; Lopez-Molina et al., 2002; Sugimoto et al., 2010). *ABI3* is an ortholog of *Viviparous-1* (*Vp-1*) in wheat, and *Vp-1* homologs play an important role in seed maturation processes such as seed dormancy and seed desiccation (McCarty et al., 1989, 1991; Giraudat et al., 1992). Mis-spliced transcripts of *TaVp-1* were identified in the A and B genomes of wheat, and different expression levels of the properly spliced transcripts of *TaVp-1A* and *TaVp-1B* were associated with different levels of seed dormancy in white-grained wheat genotypes (McKibbin et al., 2002; Yang et al., 2007a,b, 2014; Sun et al., 2012). *ABI3* encodes a transcription factor and interacts with *ABI5* to condition embryonic gene expression and seed sensitivity to ABA (Koornneef et al., 1984; Finkelstein, 1994; Lopez-Molina and Chua, 2000). *ABI5* functions as a critical factor in seed maturation and dormancy, and in dehydration tolerance of young *Arabidopsis* seedlings (Lopez-Molina et al., 2001; Sugimoto et al., 2010).

ABI5 binding protein (AFP) acts as a negative regulator in the ABA signaling pathway by facilitating degradation of ABI5 (Lopez-Molina et al., 2003). *AFP* transcription and translation increases during seed development and desiccation, ultimately reaching a plateau in mature seeds (Lopez-Molina et al., 2003; Garcia et al., 2008). Three wheat *AFP* genes (*TaAFPs*), designated *TaAFP-A*, *TaAFP-B*, and *TaAFP-D*, were isolated and localized to the short arms of chromosomes 2A, 2B, and 2D, respectively (Ohnishi et al., 2008). In our previous study, two allelic variants of *TaAFP-B* were identified and designated as *TaAFP-Ba* and *TaAFP-Bb*; *TaAFP-Ba* contained a 4 bp insertion in the promoter region compared with *TaAFP-Bb* (Feng et al., 2019). The 4 bp insertion affected mRNA stability, mRNA levels, accumulation of tdTomatoER and GUS, and GUS activity in *TaAFP-B* promoter fusions. AFPB, a co-dominant functional marker of *TaAFP-B*, was developed based on this 4 bp InDel. Mature seeds of genotype *TaAFP-B1b* generating a 203 bp fragment had stronger dormancy than the *TaAFP-B1a* genotype that produced a 207 bp fragment. The average germination indices (GI) of plants homozygous for *TaAFP-Ba* and *TaAFP-Bb* were 45.18 and 30.72%, respectively, as determined by a test of 91 white-grained Chinese wheat cultivars and advanced lines. The difference was highly significant (*P* < 0.001). Thus, *TaAFP-B* was significantly associated with PHS tolerance in common wheat (Feng et al., 2019).

In order to further elucidate the functions of *TaAFP-Ba* and *TaAFP-Bb* and investigate the mechanisms underlying seed dormancy or PHS tolerance in wheat, both alleles were transformed and investigated in rice.

## Results

### Identification and Determination of Copy Number of the *TaAFP-B* Alleles in Transgenic Rice

Twenty transgenic rice lines (T1) for each of *TaAFP-Ba*, *TaAFP-Bb*, *TaAFP-Ba-GFP* and *TaAFP-Bb-GFP* constructs were selected using hygromycin. UBF/R primers were used with the genomic DNA template from candidate lines to validate transgenic events. The frequencies of PCR positive *TaAFP-Ba*, *TaAFP-Bb*, *TaAFP-Ba-GFP* and *TaAFP-Bb-GFP* transgenic rice lines were 50.0, 70.0, 55.0, and 75.0%, respectively, for their respective transgenes (Table 1).

**TABLE 1:** Primers used for identification of transgenic plants and qPCR analysis.

Primer Set	Upstream (5′–3′)	Downstream (5′–3′)	Tm value (°C)	Fragment size (bp)
UB F/R	TTTGTTCGCTTGGTTGTGA	AGCTCGACCACCTCGTCG	58	613
Q-OsActin F/R	CCTGACGGAGCGTGGTTAC	CCAGGGCGATGTAGGAAAGC	63	321

The *hptII* (hygromycin phosphotransferase II) transgene copy numbers were determined in transgenic plants using droplet digital PCR (ddPCR) and *OsUBC* reference gene with probe labels of FAM™ and VIC™ (Tables 1, 2). The ddPCR data quality was good as indicated by the significantly different fluorescence values from negative and positive droplets, and no positive droplets were found in the negative control samples (Supplementary Figure 1). The lines had one, two, or three copies of the *hptII* transgene, and the proportions of single copy lines were 77.8, 66.7, 75.0, and 55.6% in transgenic lines with *TaAFP-Ba* and *TaAFP-Bb*, *TaAFP-Ba-GFP* and *TaAFP-Bb-GFP*, respectively. The single-copy T_1_ plants were grown to maturity and T_2_ seeds were harvested.

**TABLE 2:** Primers and probe sequences used for ddPCR.

Transgene	Primer sequence (5′–3′)	Probe sequence (5′–3′)	Tm value (°C)	Fragment size (bp)
OsUBC HptII	CCTTCGGAGACACCTTTTGA **(U)** TTGAAATGCACATTCGGGTG **(D)** GAAAAAGCCTGAACTCACCG **(U)** CATATCCACGCCCTCCTAC **(D)**	**FAM**-CTCCTTCCTCCGCAAGTTCGC-**BHQ-X** **VIC**-AAGCACGAGATTCTTCGCCC-**BHQ-X**	57 57	125 138

*Bold letters U and D mean upstream primer and downstream primer, respectively. FAM (carboxyfluorescein) and VIC (green fluorescent protein, from Aequoria Victoria) are two kinds of fluorescent report genes. BHQ-X (black hole quencher X) means a kind of quench gene.*

### Expression of the *TaAFP-B* Gene in Transgenic Rice

**FIGURE 1:**
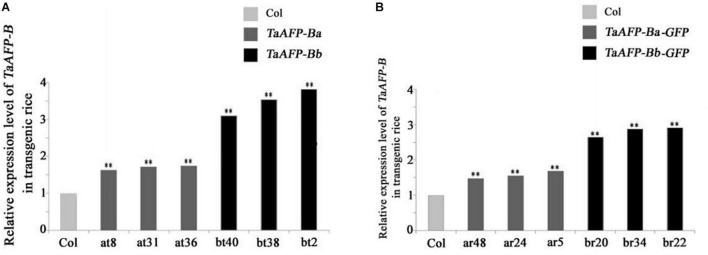
Expression levels of *TaAFP-B* in seeds of transgenic rice lines with single copies seeds of *TaAFP-Ba* (A), *TaAFP-Bb* **(A)**, *TaAFP-Ba-GFP* **(B)** and *TaAFP-Bb-GFP* **(B)**. ^**^Significant differences between transgenic plants and controls at *P* < 0.01; Col, transgenic lines with an empty vector.

**FIGURE 2:**
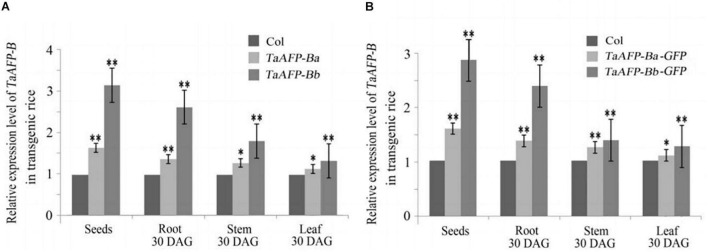
Expression levels of *TaAFP-B* in different tissues of single-copy transgenic rice of *TaAFP-Ba*, **(A)** *TaAFP-Bb*, **(A)** *TaAFP-Ba-GFP* and **(B)** *TaAFP-Bb-GFP*. **(B)** ^**^ and * significant differences between transgenic plants and controls at *P* < 0.01 and *P* < 0.05, respectively; Col, transgenic lines with an empty vector.

Because transgene copy numbers can influence expression levels, we selected only single-copy transgenic lines for further analysis. Transcript expression levels of *TaAFP-B* in seeds were measured by qPCR using an empty vector transformant as a control (Figure 1). The transcript levels of *TaAFP-Ba* and *TaAFP-Ba-GFP* were lower than those of *TaAFP-Bb* and *TaAFP-Bb-GFP* in mature transgenic rice seeds. The expression patterns and transcript levels in different tissues (roots, stems, leaves) of T2 plants 30 DAG (days after germination) and in mature seeds from T2 plants were quantified by qPCR with Q-TaAFP-B-F/R primers (Table 1). The pattern of *TaAFP-B* expression levels for all four genotypes was similar, in the order of seeds > roots > stems > leaves, and all values for transgenic plants were significantly higher than the empty vector control (Figure 2). Transcript expression levels of *TaAFP-Ba-GFP* in all four tissues were significantly lower than those of *TaAFP-Bb-GFP* (Figure 2), whereas the expression levels of *TaAFP-B* in *TaAFP-Ba* lines were slightly lower than in *TaAFP-Bb* lines (Figure 2), suggesting expression level was influenced by presence of the *GFP* reporter gene.

### *TaAFP-B* Is Induced by Abiotic Stress

**FIGURE 3:**
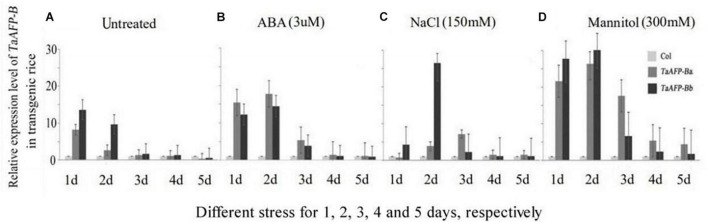
Expression profiles of *TaAFP-B* by qPCR upon exposure to different stress for 1, 2, 3, 4, and 5 days, respectively, in mature seeds of transgenic rice lines Col, transgenic lines with an empty vector. **(A)** Untreated; **(B)** ABA stress treatment; **(C)** NaCl stress treatment; **(D)** Mannitol stress treatment.

To understand how transgenic rice lines behave during abiotic stress response we examined the expression of *TaAFP-B* in the *TaAFP-Ba* and *TaAFP-Bb* lines under different stress conditions. Mature seeds from T2 plants were treated with ABA (3 mM), salt (150 mM NaCl), and mannitol (300 mM to mimic osmotic stress and dehydration) for 1–5 days. Expression levels of *TaAFP-B* in mature rice seeds with *TaAFP-Ba* were lower than those with *TaAFP-Bb* after 1 or 2 days treatment with NaCl, mannitol, and the control treated with water, whereas expression levels of *TaAFP-B* in the *TaAFP-Ba* line were higher than those in the *TaAFP-Bb* line after 1–5 days treatment with ABA, indicating that seeds of the *TaAFP-Ba* line were more sensitive to exogenous ABA than those of the *TaAFP-Bb* line. After treatment for 3 d, 4 d, and 5 d, the expression levels of *TaAFP-B* in *TaAFP-Ba* line were all higher than in the *TaAFP-Bb* line after treatment with ABA, NaCl, and mannitol, respectively (Figure 3).

### Green Fluorescent Protein Localization and Western Blotting in Transgenic Rice Carrying *TaAFP-B*

**FIGURE 4:**
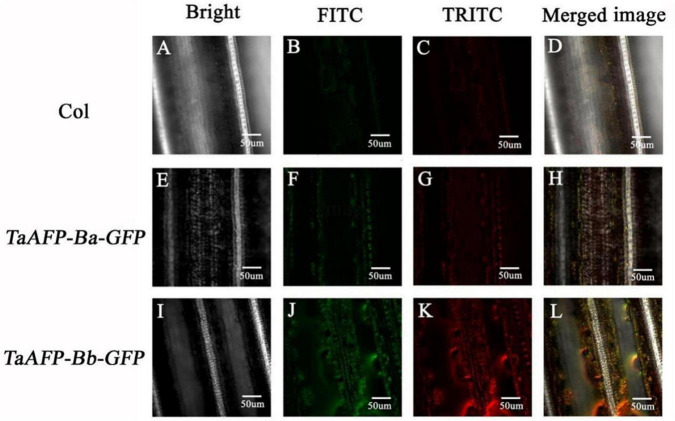
Detection of green (FITC) and red (TRITC) fluorescence in leaves of transgenic rice containing the empty vector (Col), *TaAFP-Ba-GFP* and *TaAFP-Bb-GFP* by confocal laser scanning microscopy. Magnification 40X. **(A)** Brightfield in empty vector transgenic rice leaves. **(B)** Green fluorescence in empty vector transgenic rice leaves. **(C)** Red fluorescence in empty vector transgenic rice leaves. **(D)** Merged images of **A–C**. **(E)** Brightfield in TaAFP-BaS-GFP leaves. **(F)** Green fluorescence in TaAFP-BaS-GFP leaves. **(G)** Red fluorescence in TaAFP-BaS-GFP leaves. **(H)** Merged images of **E–G**. **(I)** Brightfield in TaAFP-BbS-GFP leaves. **(J)** Green fluorescence in TaAFP-BbS-GFP leaves. **(K)** Red fluorescence in TaAFP-BbS-GFP leaves. **(L)** Merged images of **I–K**.

**FIGURE 5:**
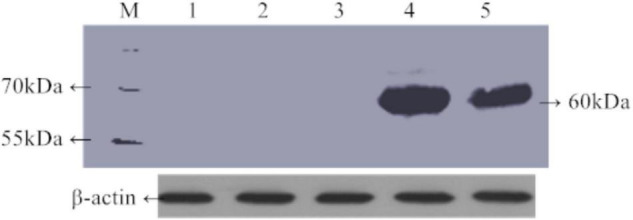
Expression level of TaAFP-B in mature seeds of transgenic rice of *TaAFP-Ba-GFP* and *TaAFP-Bb-GFP* using western blotting. M, Prestained protein marker (product#26616). 1–4: Mature seeds of 1, wild type; 2, *TaAFP-Bb* transformant; 3, *TaAFP-Ba* transformant; 4, *TaAFP-Bb*-GFP transformant; 5, *TaAFP-Ba*-GFP transformant.

Green fluorescent protein fluorescence in leaves of 30-day-old transgenic rice plants carrying *TaAFP-Ba-GFP* and *TaAFP-Bb-GFP* was visualized using confocal laser scanning microscopy. Intensity and density of GFP fluorescence was weakest in the control with the empty vector and strongest in plants with *TaAFP-Bb-GFP*. The overall trend of GFP intensity was: *TaAFP-Bb-GFP* > *TaAFP-Ba-GFP* > control (Figure 4). Western blotting analysis was performed using an Anti-GFP monoclonal antibody to detect the expression level of TaAFP-B in transgenic rice. The level of TaAFP-B-GFP fusion protein was higher than in *TaAFP-Bb-GFP* seeds than *TaAFP-Ba-GFP* seeds (Figure 5) indicating that the 4 bp insertion in the *TaAFP-Ba* promoter decreased both GFP fluorescence and TaAFP-B-GFP protein levels in mature transgenic rice seeds.

### Phenotypes of Transgenic Rice Lines

**FIGURE 6:**
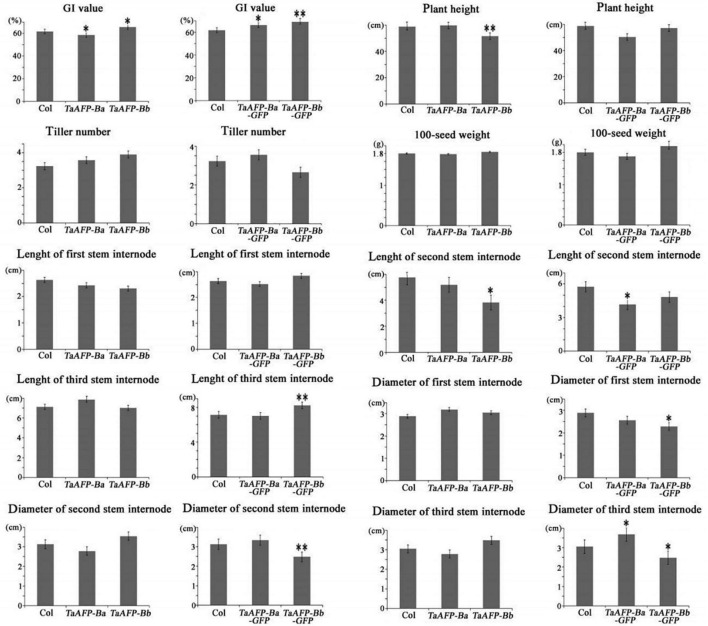
Phenotypic traits from single-copy between *TaAFP-Ba* and *TaAFP-Bb*, and between *TaAFP-Ba-GFP* and *TaAFP-Bb-GFP* transgenic rice lines. ^**^ and * mean significant differences between transgenic plants and controls at *P* < 0.01 and *P* < 0.05, respectively.

Germination index, plant height, tiller number, 100-seed weight, and length and diameter of stem internodes) of single copy T2 lines were phenotyped and analyzed (Figure 6 and Table 3). Average GI values of *TaAFP-Ba* and *TaAFP-Bb* plants were 58.5 and 65.5%, respectively, a significant difference (*P* < 0.05), compared with the control group (61.7%). The plant heights of 51.6, 59.0 and 59.8 cm for the *TaAFP-BaS, TaAFP-BbS* and control lines, respectively, showed that *TaAFP-Bb* lines were significantly shorter (*P* < 0.05). The lengths of the stem second internodes of the *TaAFP-Ba*, *TaAFP-Bb* and control genotypes were 5.2, 3.8 and 5.8 cm, respectively, with the difference between *TaAFP-Bb* genotype and control being significant (*P* < 0.05). The average GI values of *TaAFP-Ba-GFP* and *TaAFP-Bb-GFP* were 66.1 and 69.1%, respectively, both significantly different from the control (61.7%) (*P* < 0.05). The lengths of the stem second internodes of *TaAFP-Ba-GFP* and *TaAFP-Bb-GFP* were 50.5 and 57.3 cm, respectively, a significant difference (*P* < 0.01), whereas the length for the control was 59.0 cm. The diameters of the stem first, second and third internodes of *TaAFP-Bb-GFP* were 2.3, 2.5, and 2.5 mm, respectively, significantly different (*P* < 0.05) from those of the control (2.9, 3.1, and 3.1 mm, respectively). Analysis of variance indicated that differences in average GI values between *TaAFP-Ba* and *TaAFP-Bb* and between *TaAFP-Ba-GFP* and *TaAFP-Bb-GFP* genotypes were the most highly significant (*P* < 0.01) among all measured traits. These data showed that the 4 bp InDel in the *TaAFP-B* promoter affected not only average GI values in transgenic rice lines, but also internode length and diameters of all three internodes.

**TABLE 3:** Dates of phenotypic traits from single-copy between *TaAFP-Ba* and *TaAFP-Bb*, and between *TaAFP-Ba-GFP* and *TaAFP-Bb-GFP* transgenic rice lines.

	Col	*TaAFP-Ba*	*TaAFP-Bb*	*TaAFP-Ba-GFP*	*TaAFP-Bb-GFP*
GI value (%)	61.72	58.47*	65.52*	66.11*	69.08**
Plant height (cm)	58.97	59.83	51.61	50.46	57.34**
Tiller number	3.23	3.57	3.90	3.55	2.65
100-seed weight (g)	1.80	1.76	1.84	1.70	1.96
Length of first stem internode (cm)	2.63	2.43	2.31	2.52	2.84
Length of second stem internode (cm)	5.76	5.19	3.84*	4.17*	4.83
Length of third stem internode (cm)	7.12	7.85	7.03	7.02	8.21**
Diameter of first stem internode (cm)	2.89	3.19	3.05	2.56	2.28*
Diameter of second stem internode (cm)	3.13	2.78	3.54	3.34	2.48**
Diameter of third stem internode (cm)	3.05	2.78	3.48	3.68*	2.48*

** and * mean significant differences between transgenic plants and controls at P < 0.01 and P < 0.05, respectively.

**FIGURE 7:**
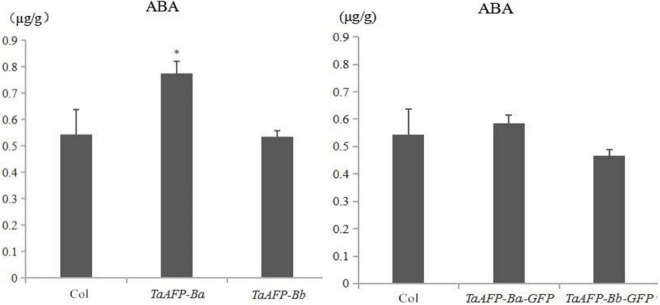
Endogenous ABA contents in mature seeds of transgenic rice lines containing *TaAFP-Ba/b*, *TaAFP-Ba/b*, *TaAFP-Ba-GFP* and *TaAFP-Bb-GFP* determined by HLPC. *Significant difference between transgenic lines containing *TaAFP-Ba* and *TaAFP-Bb* at *P* < 0.05.

Endogenous ABA content was examined in mature seeds of the *TaAFP-Ba*, *TaAFP-Bb*, *TaAFP-Ba-GFP* and *TaAFP-Bb-GFP* lines by HPLC (high performance liquid chromatography). The average values of endogenous ABA in seeds of the *TaAFP-Ba* and *TaAFP-Bb* lines at 0.8 and 0.5 μg/g, respectively, were significantly different (*P* < 0.05), and the value for the *TaAFP-Ba-GFP* line (0.6 μg/g) was also higher than that of the *TaAFP-Bb-GFP* line (0.5 μg/g) (Figure 7). Thus, the 4 bp insertion in the *TaAFP-Ba* promoter led to a higher endogenous ABA content in mature seeds of transgenic rice.

## Discussion

Transcript levels of the *TaAFP-Ba* allele were markedly higher than those of the *TaAFP-Bb* in different tissues and were correlated with the observed GUS activity in *ProTaAFP-Ba:GUS* transgenic rice (Feng et al., 2019). Those results suggested that the 4 bp InDel in the 5′UTR of *TaAFP-B* affected gene expression and function. The present analysis showed (1) that transcript levels of *TaAFP-B* were lower in transgenic rice lines containing *TaAFP-Ba* and *TaAFP-Ba-GFP* than in lines with *TaAFP-Bb* and *TaAFP-Bb-GFP* (Figure 2); (2) that the GFP fusion protein levels in seeds of lines containing *TaAFP-Ba-GFP* was lower than in those with *TaAFP-Bb-GFP* (Figures 4, 5); and (3) that average GI values of lines with *TaAFP-Ba* and *TaAFP-Ba-GFP* lines were lower than those of lines containing *TaAFP-Bb* and *TaAFP-Bb-GFP* (Figure 6). Taken together, these results showed that the higher transcript expression and protein levels of *TaAFP-Bb* led to higher GI values in mature transgenic rice seed. Thus, the 4 bp InDel in the 5′UTR of *TaAFP-B* had a significant effect on expression level of *TaAFP-B* which, in turn, influenced the GI value of mature seeds and several other traits.

ABA plays an important role in seed maturation and dormancy (Gubler et al., 2005; Nakashima et al., 2006). Accumulation and activity of *AFP* in *Arabidopsis* are induced by ABA during seed germination (Lopez-Molina et al., 2002). It was shown that a negative correlation is present between the germination ability of embryos and the sensitivity of embryos to ABA. Moreover, there is evidence that ABA levels in mature wheat embryos are similar in both PHS-sensitive and -resistant cultivars, and that sprouting behavior is related more to the extent of ABA sensitivity of embryos than to actual endogenous ABA content (Walker-Simmons and Sesing, 1990). In this study, the transcript expression levels of *TaAFP-B* in mature seeds of transgenic lines carrying *TaAFP-Ba* and *TaAFP-Bb* were determined following treatment with ABA. Our results indicated that *TaAFP-B* transcript expression levels in the transgenic lines had different sensitivities to ABA (Figure 3) that not only affected dormancy but also plant height and length and diameter stem internodes. The 4 bp InDel in the promoter of *TaAFP-B* not only affected the mRNA transcription level, mRNA decay, translation levels of GUS, tdTomatoER, and GUS activity in wheat (Feng et al., 2019), but also transcript and translation levels of *TaAFP-B*, sensitivity to ABA, content of endogenous ABA, and average GI values in mature seeds of transgenic rice. The allelic variation of *TaAFP-B* was markedly associated with seed dormancy and therefore pre-harvest sprouting tolerance in wheat. Furthermore, the 4-bp InDel in the promoter of *TaAFP-B* also affected markedly the plant height, lengths of the second and third internodes, diameters of the first to third internodes, but these values of phenotypes have not a same trend between transgenic rice plants of *TaAFP-Ba* and *TaAFP-Bb*, and between *TaAFP-Ba-GFP* and *TaAFP-Bb-GFP* (Figure 6), which may be a reason of GFP fusion protein affecting these phenotypes. In addition, the change in sensitivity to ABA in *TaAFP-Ba* transgenic rice might also be responsible for changes of lengths and diameters of the second and third stem internodes and plant height in transgenic rice, because ABI5 is a key positive factor in ABA signaling pathway in plants, which has the capability of binding the *SbGA2ox3* 5′-regulatory region and promote *SbGA2ox3* protein accumulation, resulting in degradation of GA4 in sorghum (Renata et al., 2013); in addition, during phyB-dependent inhibition of germination in Arabidopsis, AtABI5 interacts with *AtGA3ox1* and *AtGA3ox2*, but suppresses their expression instead, which results in lower GA levels in the seed (Lee et al., 2012). These proofs of ABI5 function showed a cross-talk between function of ABI5, ABA signaling and GA metabolism, corroborating the fact that *TaAFPs* were orthologs of *AtAFP* (Ohnishi et al., 2008), so it is deduced that there is also indirect relationship between function of TaAFP, ABA signaling and GA metabolism. The change in sensitivity to ABA in *TaAFP-Ba* transgenic rice might lead to change of GA content and further affect the lengths and diameters of the second and third stem internodes and plant height in transgenic rice, but these requires further investigation.

There was a difference in transcript levels of *TaAFP-Ba* and *TaAFP-Bb* in common wheat and transgenic rice. In a previous study in common wheat (Feng et al., 2019), trend of transcript levels of *TaAFP-B* in seeds at different days after pollination was always *TaAFP-Ba* > *TaAFP-Bb* compared with *TaAFP-Bb* > *TaAFP-Ba* in the present work with transgenic rice. The reasons for the different trend of transcript expression levels of *TaAFP-Ba* and *TaAFP-Bb* between wheat and transgenic rice might be associated with different genetic backgrounds of these two species or result of *TaAFP-B* overexpression. Moreover, the transcript levels of *TaAFP-B* in *TaAFP-Ba* transgenic rice seeds treated with ABA were more sensitive than those in the *TaAFP-Bb* genotype (Figure 3). Endogenous ABA content in mature seeds of the transgenic lines measured by atomic absorption showed that the endogenous ABA content was higher in mature seeds containing *TaAFP-Ba* and *TaAFP-Ba-GFP* than those containing *TaAFP-Bb* and *TaAFP-Bb-GFP* (Figure 7). It can therefore be deduced that the level of endogenous ABA in mature seeds of transgenic rice had been altered by overexpression of *TaAFP-B*. *ABI5* is a positive regulatory factor in the ABA signaling pathway (Lopez-Molina et al., 2001) and regulates the maturation and germination of seeds at later stages of maturity (Lopez-Molina et al., 2001, 2002; Carles et al., 2002), and AFP is a novel negative regulator of ABA signaling that attenuates ABA signaling by targeting ABI5 for ubiquitin-mediated degradation in nuclear bodies (Lopez-Molina et al., 2003). The higher level of endogenous ABA in mature seed of the transgenic line with *TaAFP-Ba* is attributed to less *TaAFP-B* transcript and less TaAFP-B protein that lead to higher seed dormancy.

### Conclusion

The 4 bp insertion in the promoter of *TaAFP-Ba* decreased the transcript expression and translation level in transgenic rice. Increased endogenous ABA content in mature seeds and affected their sensitivity to ABA leading to higher seed dormancy and PHS tolerance, and also effected the diameter and length of the second and third internodes and plant height. These results provide evidence for potential application of the allelic variation in *TaAFP-B* for improvement of PHS and lodging tolerance in wheat.

## Materials and Methods

### Plasmid Construction

The pCAMBIA1390-Ubi-GFP vector was kindly supplied by Professor Lanqin Xia, Institute of Crop Sciences, Chinese Academy of Agricultural Sciences (CAAS). The sequences of *TaAFP-Ba* and *TaAFP-Bb* were 1,058 bp (105 bp fragment of 5′UTR and 953 bp CDS) and 1,054 (101 bp fragment of 5′UTR and 953 bp CDS) bp, respectively (the difference due to a 4 bp InDel in the 5′UTR). The sequence of the *TaAFP-B* alleles were amplified and cloned into vectors pCAMBIA1390-Ubi and pCAMBIA1390-Ubi-GFP by General Biosystems (Anhui) Co., Ltd., respectively.

### Transformation of Rice Plants

*Agrobacterium tumefaciens* strain EHA105 was used to genetically transform embryonic calli of rice (*Oryza sativa* L. ssp. japonica cultivar Nipponbare, kindly supplied by Professor Lanqin Xia, CAAS) with previously described recombinant vectors (Feng et al., 2019). Transformed calli were screened and planted on regeneration medium under hygromycin selection (50 mg/L) to acquire T_1_ plants.

### Identification of Transgenic Plants and Copy Number Determination

All transgenic rice lines were planted in a greenhouse and genomic DNA was isolated from bulked young leaf samples from 5 plants of each T_1_ line according to the guidelines for the DNA Secure Plant Kit (TIANGEN Biotech). PCR was performed as described previously to identify positive transgenic lines (Feng et al., 2019). Amplified PCR fragments and primer sets are listed in Table 1. The quality of genomic DNA was determined using a BioDrop spectrophotometer, and copy numbers were detected using ddPCR (Collier et al., 2017, Shanghai Biotechnology Co., Ltd.). A primer pair and probe were designed to detect a unique single-copy insertion for target and endogenous reference genes, respectively. The target and endogenous reference genes were *hptII* and *OsUBC*, respectively (Table 2). Reference gene amplification was detected using a FAM™-labeled probe whereas target gene amplification was detected with the VIC™-labeled probe. The ddPCR reaction mixture consisted of 2 μL DNA, 10 μL of 1X QX200 EvaGreen Supermix (Bio-Rad), 450 nM of each primer pair, and 250 nM of each probe. Thermal cycling conditions were 95°C for 10 min, followed by 40 cycles of 94°C for 30s and 60°C for 30s, 98°C for 10 min, and a final hold at 4°C. The detailed experimental procedures were referenced from McCord (2016) and Collier et al. (2017). We planted and identified at least three positive independent single-copy lines for each transgenic type. Plants for subsequent analysis were grown at 25°C under 16/8 h light/darkness in a greenhouse at the Anhui Academy of Agricultural Sciences Base for transgenic rice.

### Transgenic Plant Phenotyping

The single-copy positive plants of each transgenic rice line were selected for phenotypic analysis after qPCR. The T2 generations, 42 plants from three single-copy positive lines of transgenic rice of *TaAFP-Ba*, *TaAFP-Bb*, *TaAFP-Ba-GFP* and *TaAFP-Bb-GFP*, respectively, were analyzed for germination index, plant height, tiller number, 100-seed weight, and stem length diameter.

### Gene Expression Analysis

Total RNA was extracted from 5 g samples of roots, stems, leaves and seeds, respectively, with the TaKaRa MiniBEST Plant RNA Extraction Kit (TaKaRa). First-strand cDNA was synthesized and qPCR was carried out using a LightCycler^®^480 System Real-Time PCR as described by Feng et al. (2019). All primers for the target genes are listed in Table 1. Relative expression of target genes was evaluated by the 2^–ΔΔCt^ method (Li et al., 2018). The experiments were conducted in three biological replications.

### Germination Assays and Stress Treatments

The seeds from single-copy T_2_ lines were sterilized in 75% ethyl alcohol and were sown in a growth chamber with a 16/8 h light/darkness cycle at 28°C. For germination assays, 100 seeds were germinated and grown on solid 1/2-MS medium for 7 days. The GI value for each transgenic line were calculated according to the number of germinated seeds on a daily basis (Walker-Simmons, 1988). For stress treatments, the seeds were incubated on solid 1/2-MS medium including 3 μM ABA, 150 mM NaCl (for salt stress), or 300 mM mannitol (for dehydration stress), respectively, for 1, 2, 3, 4, and 5 days. Transgenic seeds from empty vector transformants was used as controls and each experiment included three biological replicates. All samples were collected after each treatment and frozen in liquid nitrogen and stored at −80°C.

### Fluorescent Protein Expression and Western Blotting

FITC (fluorescein isothiocyanate) and TRITC (tetramethylrho damine) filters were used to assess green fluorescence for GFP and red fluorescence for chlorophyll autofluorescence from chloroplasts. The fluorescence intensity of merged images with orange color revealed the expression of GFP. We selected young leaves of single copy positive plants at 30 days after germination (DAG) to analyze the expression of GFP in transgenic rice with *TaAFP-Ba-GFP* and *TaAFP-Bb-GFP* constructs mounted on covered glass slides by an Olympus BX-60 of Confocal Laser Scanning Microscope. We used excitation lasers at 488 and 568 nm to excitate GFP and chlorophyll, respectively; images were collected by FITC and TRITC filters, and single-channel images were superimposed to observe the expression of GFP in leaves. At the same time, Anti-GFP (TransGen Biotech) was selected as a probe to detect the TaAFPB fusion protein in transgenic rice.

## Data Availability Statement

The original contributions presented in the study are included in the article/Supplementary Material, further inquiries can be directed to the corresponding author/s.

## Author Contributions

YF and YH performed the experiments and wrote the manuscript. BH assisted in performing experiments. YZ planted the experimental materials and determined the GI values. YY and YX designed the experiments and assisted in writing the manuscript. All authors have read and approved the final manuscript.

## Conflict of Interest

The authors declare that the research was conducted in the absence of any commercial or financial relationships that could be construed as a potential conflict of interest.

## Publisher’s Note

All claims expressed in this article are solely those of the authors and do not necessarily represent those of their affiliated organizations, or those of the publisher, the editors and the reviewers. Any product that may be evaluated in this article, or claim that may be made by its manufacturer, is not guaranteed or endorsed by the publisher.

## Abbreviations

PHS: pre-harvest sprouting
ABA: abscisic acid
GA: gibberellins
*Vp-1*: *viviparous-1*
LEA: late embryogenesis abundant
InDel: insertion-deletion
UTR: untranslated region
GFP: green fluorescent protein
FITC: fluorescein isothiocyanate
TRITC: tetramethylrhodamine
HPLC: high performance liquid chromatography.
