# Supplementary figures and images for: A 4 bp InDel in the Promoter of Wheat Gene TaAFP-B Affecting Seed Dormancy Confirmed in Transgenic Rice
Source: Front Plant Sci. 2022 Mar 31;13:837805. doi: 10.3389/fpls.2022.837805 (PMC9008840; doi:10.3389/fpls.2022.837805)

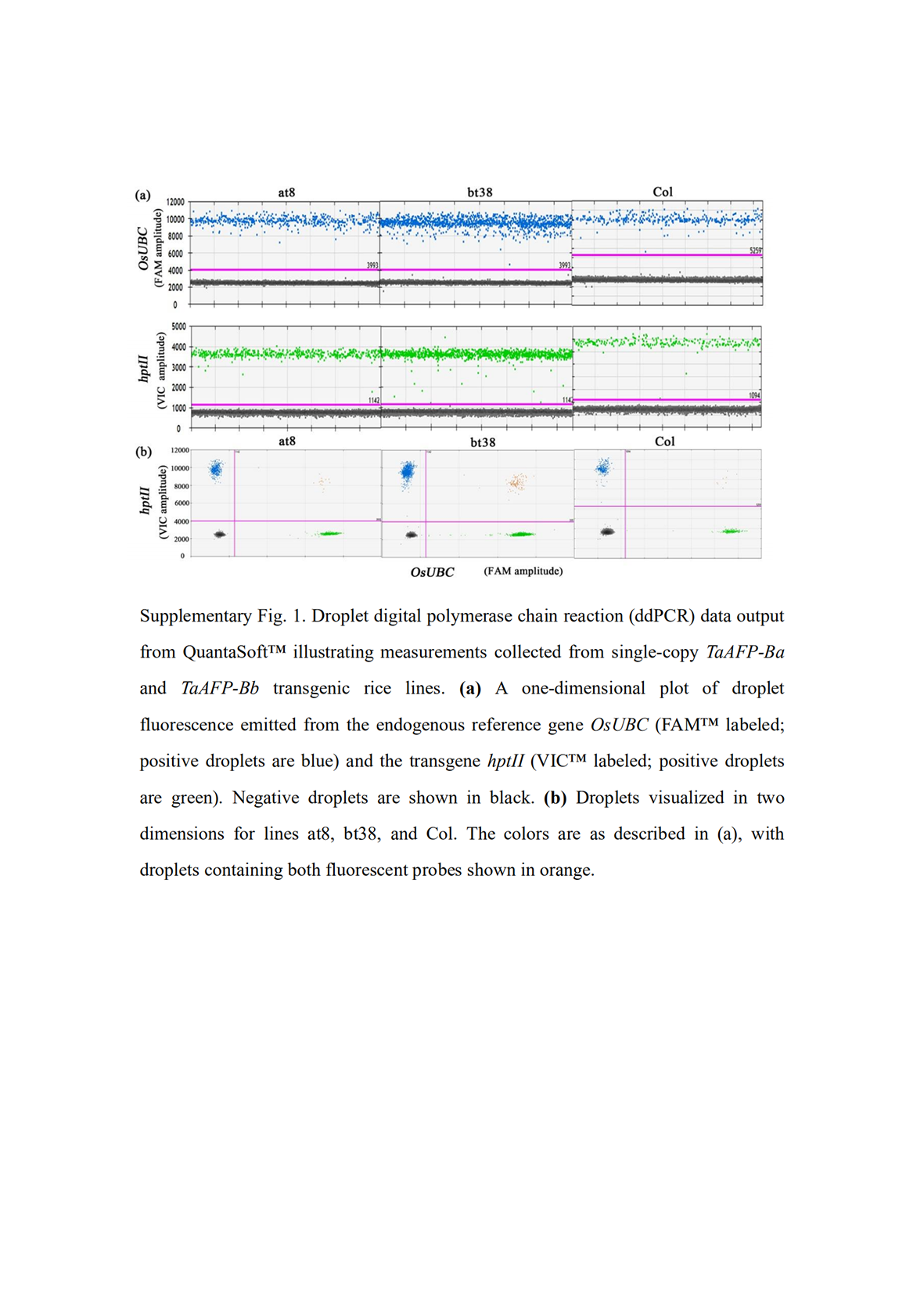

Supplement: Supplementary file 1 [file Image_1.TIF]
